# Bio-interfacial engineering of electrophoretic coacervate microcapsules for cytocompatible on-skin displays

**DOI:** 10.1098/rsif.2026.0105

**Published:** 2026-09-09

**Authors:** Wenda Zhao, Daniele Baiocco, Asme Boussahel, Liam Grover, Zhibing Zhang, Anne Roudaut

**Affiliations:** ^1^ Department of Computer Science, University of Bristol, Bristol, UK; ^2^ School of Biochemistry, Biomedical Science Building, University of Bristol, Bristol, UK; ^3^ School of Computer Science, University of Bristol, Bristol, UK; ^4^ School of Chemical Engineering, University of Birmingham, Birmingham, UK; ^5^ Department of Chemical Engineering, School of Aeronautical, Automotive, Chemical and Materials Engineering, Loughborough University, Loughborough, UK

**Keywords:** e-ink microcapsules, electrochromic displays, on-skin digital devices, flexible electronics, micromanipulation, biocompatibility graphical

## Abstract

Electrophoretic ink (e-ink) holds significant potential for energy-efficient displays and sustainable alternatives to print media. However, their use in wearables is hindered by hazardous materials which are cytotoxic and sensitizing to human tissue. While commercial e-inks encapsulate these fluids in rigid, synthetic microplastics, such as melamine-formaldehyde, they lack the mechanical conformability and environmental safety required for epidermal contact. We thus present a novel approach for producing e-ink microcapsules (EIMCs) for integration into biocompatible and free-form digital devices. Spherical, core–shell EIMCs were fabricated via complex coacervation (pH 4.1), using user-friendly biopolymers (gum acacia and gelatin). A core of white electrophoretic TiO_2_ particles (diameter approx. 30 nm) was marked with a darker violet dye, while being homodispersed in hexylsalicylate-tetrachloroethylene (HS-PCE), as the liquid medium. Characterization of the physico-chemical and performance properties of EIMCs showed remarkable nominal compression stress at rupture (3.9 ± 0.9 MPa). When subjected to a DC voltage of 20 V, the microcapsules created a writable display, potentially enabling users to write and rewrite. The response time was approximately 0.5 seconds (2 Hz refresh rate), comparable to commercial products. *In vitro* cytotoxicity and CD54 expression (h-CLAT) assays demonstrated that the microcapsule shell successfully mitigated solvent-induced immunotoxicity, maintaining high cellular viability (approx. 70%) and minimal sensitization (approx. 1.7%).

## 1. Introduction

Electronic papers have emerged as a revolutionary, sustainable display technology, offering bistability, flexibility and ultra-low power dissipation, with their global market projected to reach beyond $5.5 billion by 2030 [1,2]. Unlike conventional emissive screens, they mimic the optical properties of traditional ink on paper, which is leveraged by the presence of an electrophoretic ink [3]. At the heart of the e-ink system lies the microcapsules in which charged pigmented particles (CPPs) are entrapped while suspended within a colourless dielectric liquid medium. When an electric field is applied, CPPs move negentropically, yielding changes in microcapsule coloration [4].

Historically, the fabrication of electrophoretic microcapsules has relied on synthetic polymer shells and industrially engineered pigments [3]. While these materials provide durability, they pose challenges to broader-spectrum applications owing to insufficient contrast and slow response speed, alongside concerns regarding their environmental sustainability, cost-effectiveness and manufacturing complexity [5]. More critically, for emerging wearable electronics, the shell of the electrophoretic microcapsule no longer acts solely as a passive CPP-retaining barrier, but rather as the primary biological interface between electrophoretically active fluids and human tissue. As such, shell composition and interfacial engineering become central determinants of both electrophoretic functionality and biological safety.

Dyes and pigments play a pivotal role in the optical properties of electrophoretic displays. Titanium dioxide (TiO_2_) has been widely adopted owing to its superior reflectivity and brightness. However, its high specific density (approx. 4 g cm^−^^3^), and thus its tendency to sediment, introduce hurdles in maintaining long-term suspension stability [6,7]. Darker pigments, such as carbon black and phthalocyanine-based dyes, provide strong contrast but require heavy surface modifications to ensure optimal electrophoretic behaviour [7]. Strategies to enhance white pigment performance are being explored, with special emphasis on particle size reduction, charge-control additives and polymeric coatings to ease the density mismatch between the dielectric medium and titanium dioxide [4,7]. The dielectric medium should facilitate the movement of CPPs, ensuring rapid response to the applied voltage. However, commonly used aliphatic hydrocarbons or silicone-based media face shortcomings in viscosity and environmental sustainability [3]. Nevertheless, the trade-off among CPP stability/mobility, highly performing dielectric medium and optical clarity remains an ongoing technical challenge towards enhancing display longevity and performance.

Beyond conventional e-paper applications, wearable on-skin displays are transforming biomedical research by providing unique platforms for health monitoring, diagnostics and therapeutic applications [8]. A critical component of these devices is the display material, which enables dynamic data streaming directly on the skin [9]. While electrophoretically active e-ink microcapsules (EIMCs), with a shell made of melamine/urea-formaldehyde [10], have been proposed for e-papers, their adaptation for on-skin biomedical devices faces challenges, including poor microcapsule suspendability, impaired biocompatibility and/or pigment clumping [3,11]. Therefore, the microcapsule shell material is the critical biological interface, which influences the stability and electrophoretic performance of EIMCs, provides mechanical strength, while mitigating any related human immunogenic response (e.g. skin sensitization).

Traditionally, the fabrication of electrophoretic microcapsules relies on petroleum-sourced polymers, such as polyurea [12], urea-formaldehyde [4] and melamine-formaldehyde, featuring poly(methyl methacrylate) (PMMA) [13], but they pose severe limitations for wearable applications. They lack the mechanical compliance required to withstand dynamic human joint deformation, and their synthetic nature raises stringent regulatory and biocompatibility concerns for prolonged skin contact [14,15]. Consequently, ideal encapsulation for on-skin devices requires sustainable materials that provide high mechanical resilience and electrical insulation without relying on immunogenic synthetic precursors [11]. To this end, biopolymeric encapsulation using gelatin and gum acacia presents a promising approach [16,17], offering biodegradability, interfacial stability, mechanical strength, and cost-effectiveness while preserving the functional integrity and electrophoretic activity of the microcapsules [18].

Recent work has begun to explore this direction within the human–computer interaction (HCI) community, where the challenge of democratizing access to e-ink microcapsules has been identified as an important step towards enabling new forms of interactive display fabrication [7,11]. That work introduced a simplified coacervation-based process to synthesize electrophoretically active capsules and highlighted its potential for design-oriented prototyping. But it addressed accessibility from a fabrication perspective, with limited attention to deeper materials analysis or biomedical applicability [19]. In contrast, the present work advances scientific understanding on the bio-interfacial properties provided by the coacervate shells towards effective cytocompatibility, immune shielding and compliant integration with epidermal systems.

Herein, we present an engineering strategy to overcome this electric and cytotoxicity trade-off. We demonstrate that core-shell biopolymer microcapsules, fabricated via complex coacervation of gelatin and gum acacia, can be cross-linked to act as a highly impermeable immune-toxicological shield. Rather than merely containing the ink, this biopolymer architecture is deliberately engineered to segregate a highly active, immunogenic electrophoretic core (comprising highly charged, surface-modified TiO_2_ nanoparticles and gentian violet in a hexylsalicylate-tetrachloroethylene (HS-PCE) medium) from the surrounding biological environment. We detail the solid–liquid interfacial phenomena driving nanoparticle stability, the superior mechanical resilience of the cross-linked coacervate shell under compression, and explicitly validate its biological safety via rigorous *in vitro* cytotoxicity and CD54 expression (human cell line activation test; h-CLAT) assays. Ultimately, this approach establishes a robust framework for safely integrating high-performance electrophoretic fluids into flexible, human–machine interfaces.

## 2. Results

All experiments were performed in triplicate unless stated otherwise. Data variability is expressed as the mean value ± standard error (s.e.).

### 2.1. E-ink microcapsules scanning electron microscopy observation

**Figure 1.**
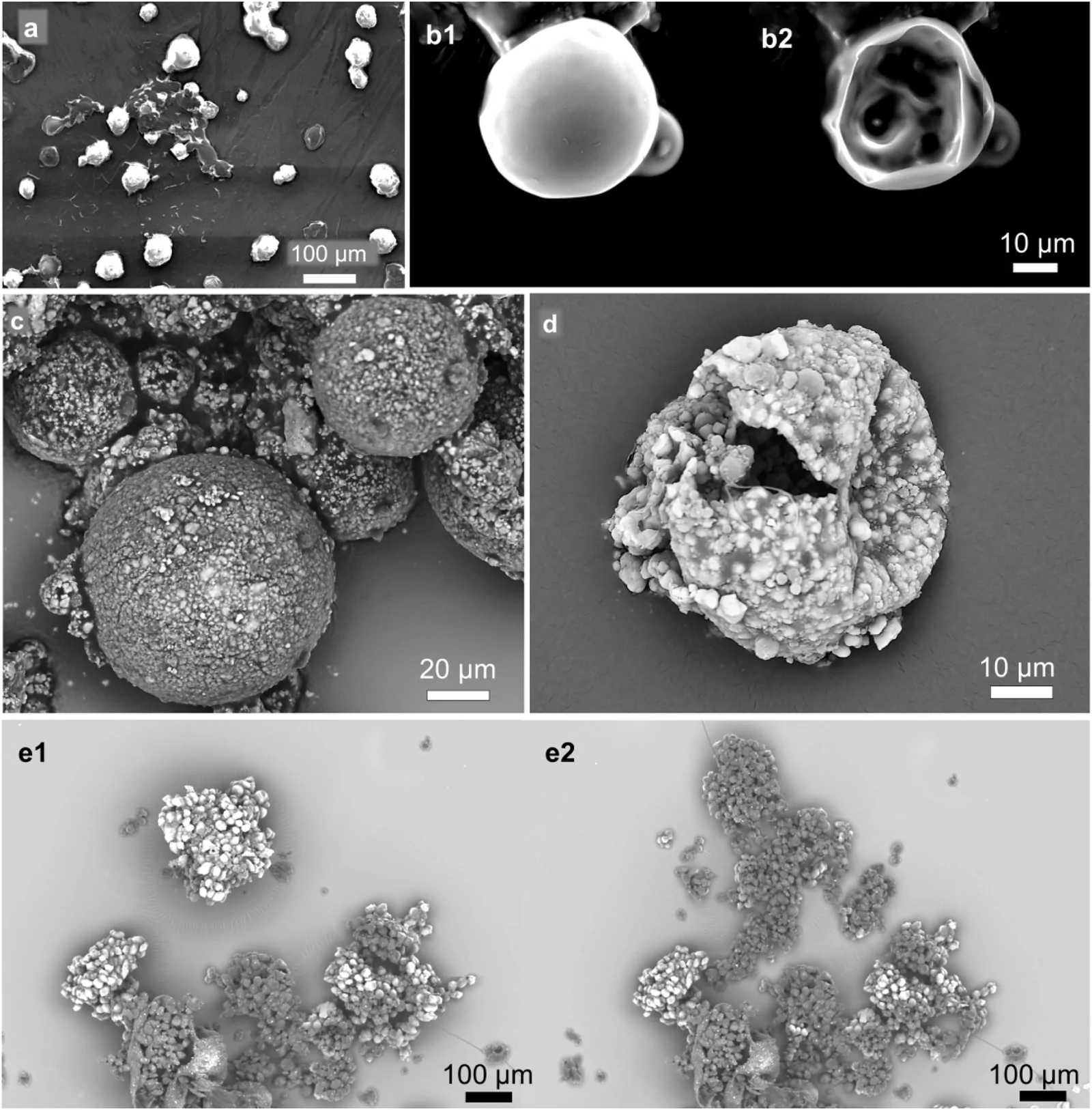
SEM images of EIMC microcapsules highlighting their morphology and surface characteristics: (a) overview of heterodisperse cross-linked microcapsules with a relatively spherical morphology; close-up of (b1) an intact spherical microcapsule with a smooth outer shell (b2) after bursting, revealing its hollow core-shell structure; (c) microcapsule with a relatively rough surface owing to embedded TiO_2_ nanoparticles, deploying at its surface; (d) damaged microcapsule with visible cracks confirming a core–shell structure; SEM micrographs of electronic ink microcapsules before (e1) and after (e2) exposure to a 15 kV electron beam for over 120 s. Post-exposure, significant microparticle rearrangement is observed, probably owing to surface charging and thermal effects, triggering chromatic response of the ink system.

Both morphological and surface topographical properties of cross-linked EIMCs (*D*_nb,J_ = 22.4 ± 0.4 µm; *n* = 200; supplementary material, table S1) were investigated by scanning electron microscopy (SEM). Figure 1a presents an overview of heterodisperse EIMC microcapsules, predominantly exhibiting a spherical morphology. While the microcapsules appear self-isolated, some areas show signs of agglomeration, probably owing to the presence of the cross-linking agent. This enhances the structural integrity of the EIMCs, preventing premature degradation in the vacuumed SEM chamber, but may also induce colloidal aggregation, leading to capsule-to-capsule bridging/gluing phenomena [20].

A close-up of a spherical microcapsule with a core–shell configuration is shown in figure 1b. The smooth outer shell in figure 1b1 indicates an intact structure, essential for effectively encapsulating the electrophoretic core. In contrast, figure 1b2 depicts a partially damaged or ruptured capsule, probably owing to prolonged exposure to the SEM electron beam under high vacuum conditions, at a reduced working distance (10 mm) and a relatively high accelerating voltage (15 kV) [21]. This damage confirms the internal hollow structure of the EIMC. For electrophoretic applications, the core–shell configuration is a desirable design, ensuring effective containment of the electrophoretic medium while providing mechanical and structural stability, which is essential for long-term performance in display or optical applications [22].

The micrograph in figure 1c depicts a microcapsule with a rough, textured surface, probably resulting from the inhomogeneous adhesion of TiO_2_ nanoparticles (NPs) onto the shell [23]. The uneven surface morphology suggests that these NPs may have been embedded during the encapsulation process or attached post-formation owing to electrostatic interactions, van der Waals forces or capillary-driven deposition [24].

In figure 1d, a fractured microcapsule is shown, exhibiting visible cracks across its surface. The rupture probably resulted from a combination of electromechanical and thermal stress, induced by continuous exposure to the electron beam and vacuum pressure during SEM imaging [25]. This also appears to have led to the vaporization of the liquid electrophoretic content. Yet, the modified TiO_2_ NPs are possibly accumulating near the cracked regions. Energy dispersive X-ray spectroscopy (EDX) analysis of the white clump on the uneven surface confirmed the presence of TiO_2_, with a high atomic concentration of titanium (approx. 17%). This concentration increases to over 50% when measured on the inner side of the crack (supplementary material, figure S4, table S2), where the density of nanoparticles is highest, as expected. Overall, the incorporation of TiO_2_ NPs within the shell matrix is conducive to the overall structural integrity of the microcapsules and may contribute to their functional properties, such as improved mechanical strength and stability, and optical performance [26,27].

In figure 1e, the SEM micrographs depict EIMCs, containing violet dye in conjunction with TiO_2_ NPs, before (e1) and after (e2) exposure to a 15 kV electron beam for a relatively prolonged period (greater than 120 s). The images reveal a distinct rearrangement of microparticles following electron beam exposure, indicative of potential physical changes. Specifically, in figure 1e1, the microcapsules exhibit a compact and aggregated morphology, with well-defined clusters. TiO_2_ NP-dominated regions appear bright, densely embedded within the microcapsule structures. Following exposure to the electron beam (figure 1e2), significant rearrangement of the microparticles was observed. This probably resulted from localized charging effects, thermal energy or alterations in interparticle adhesion owing to the high-energy electron interactions [25,28]. The redistribution of EIMCs suggests potential surface charging may be triggered by electrostatic effects, thereby perturbing the quiescence of the encapsulated ink.

Interestingly, the microcapsule morphology remained largely intact, despite the redistribution of EIMC, which appeared darker and more randomly spread out (figure 1e2). This provides clear evidence of their effective chromatic response.

This observation underscores the sensitivity and dynamic behaviour of EIMCs under electron beam exposure, with potential implications for their structural stability in electron-rich/high-energy environments (greater than 15 kV), under prolonged operations. However, it is important to note that typical e-paper and e-display technologies operate at no/minimal voltages (0–20 V), unveiling the potential of our EIMCs for practical applications [29].

### 2.2. Applied electric field: observation of e-ink microcapsules

**Figure 2.**
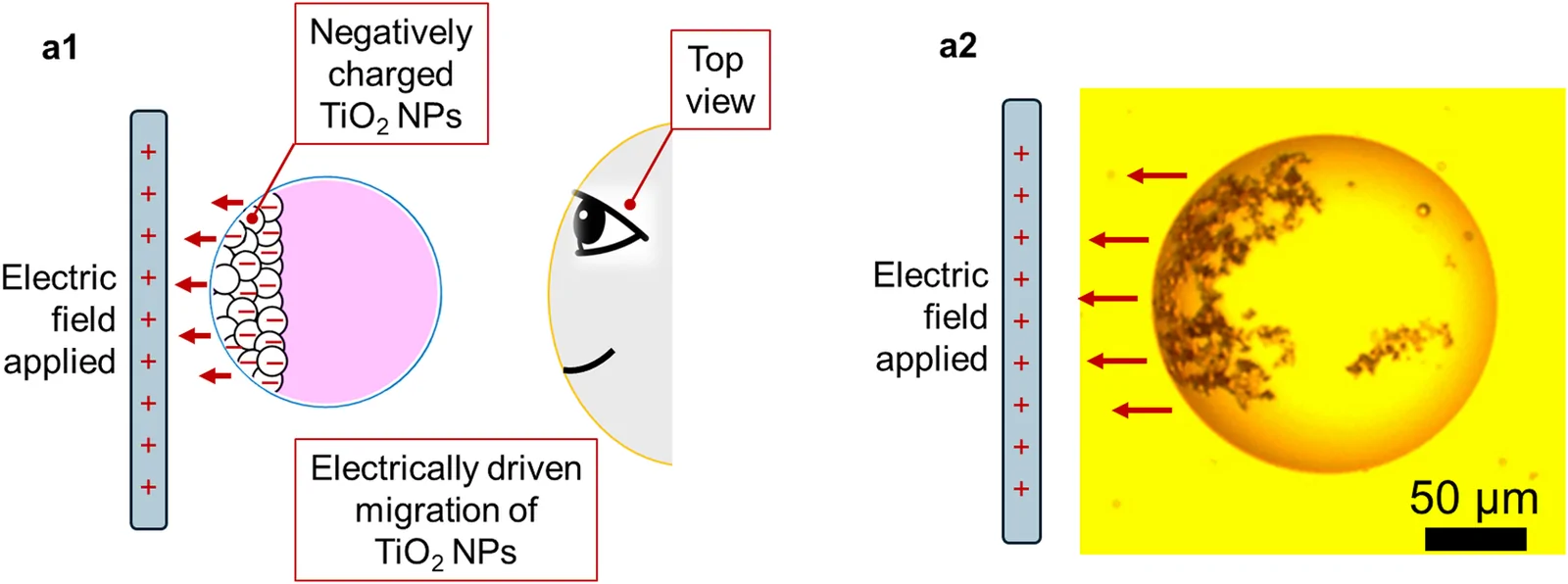
Electrophoretic behaviour of TiO_2_ NPs within microcapsules under an applied electric field: (a1) schematic representation of TiO_2_ NP migration toward the positively charged electrode, forming a dense layer along the inner shell of the microcapsule; (a2) real-time microscopic observation demonstrating the rapid response (less than 0.5 s) of NPs, where densely packed regions block light transmission, while particle-depleted areas remain transparent. This behaviour underpins the electrophoretic principle in electronic e-ink technology.

Upon applying an electric field, the negatively charged TiO_2_ NPs suspended within the dielectric medium exhibited an instantaneous response, migrating toward the positively charged electrode (figure 2). Figure 2a1 schematically represents the fundamental principle, illustrating how the NPs concentrate near the oppositely charged electrode, forming a dense layer stacking along the curvature of the microcapsule inner shell. This migration follows the electrostatic force lines, which are maximized in regions where the electrode–particle optical pathway is minimized. The real-time microscopic observation shown in figure 2a2 provides direct evidence of this rapid response, occurring within a timeframe of less than 0.5 s. It became evident that the accumulation of TiO_2_ NPs effectively blocked light transmission in regions where they are densely packed, while areas devoid of particles remained transparent. Indeed, this behaviour is pivotal to electrophoretically activated e-ink technologies, where nanoparticle displacement alters the optical appearance of the microcapsule [3]. Key insights are the curvature of the microcapsule inner shell and its structural configuration (core–shell), which played a crucial role in guiding NP migration. Conversely, irregularly shaped and/or multinuclear microcapsules may alter the dielectric properties of the suspension medium, impairing the response speed and/or contrast ratio in practical applications.

### 2.3. Actuation tests: electrophoretic writing and display applications

**Figure 3.**
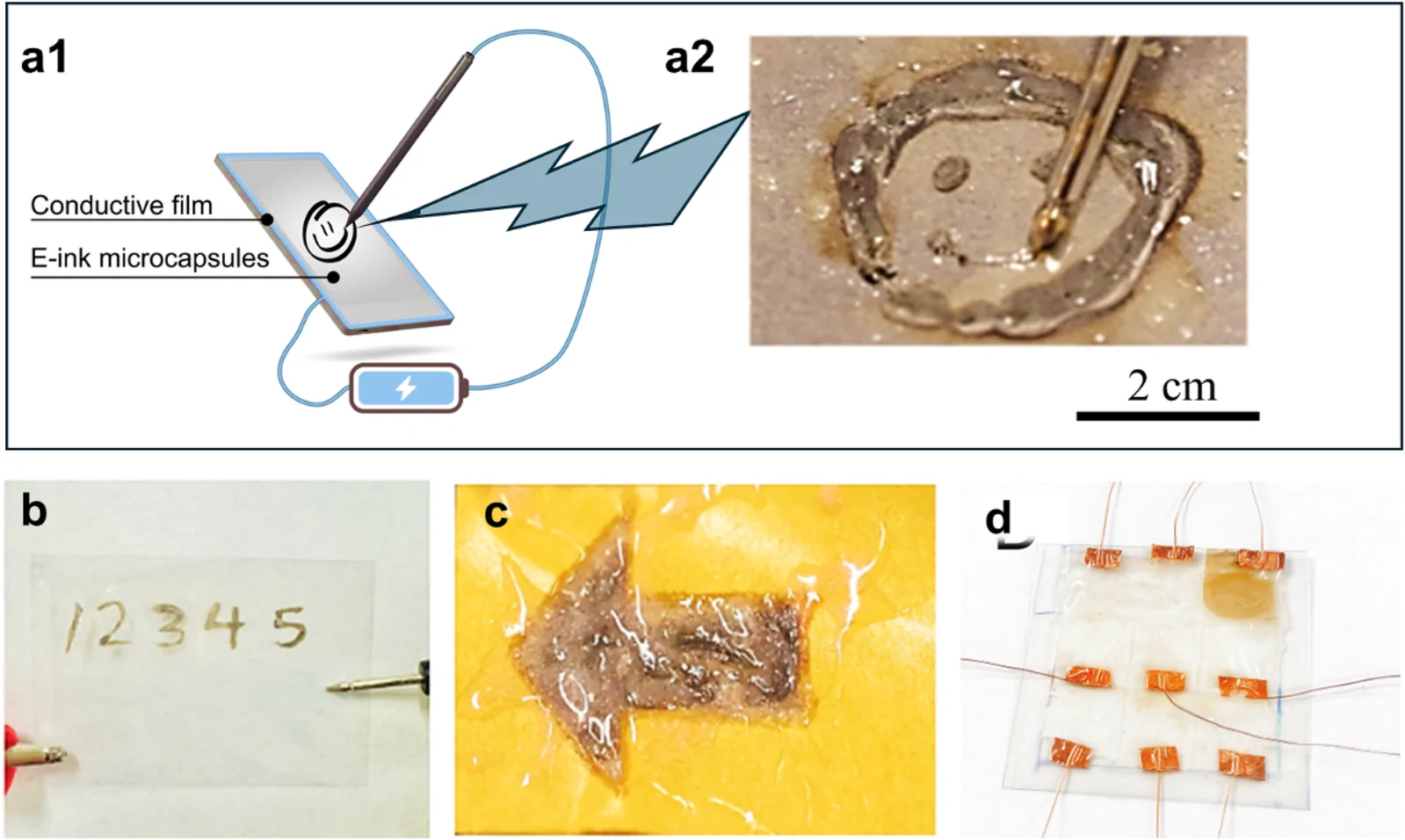
Electrophoretic freehand e-ink writing and display applications: (a1) schematic of the writing display, where a stylus connected to a power source modulates the electric field, redistributing NPs to create visible emoji (a2); (b) practical demonstration of the system, showing the inscription of numbers (e.g. 1, 2, 3, 4, 5) with high contrast and rapid response; (c) stencilled shape with predefined patterns (e.g. arrow) toggled on and off using controlled electrical stimulation; (d) a rudimentary 3×3 matrix display, demonstrating individually addressable cells for differential data streaming (e.g. digital indicators).

In figure 3, the principle of a dynamic, electrophoretically driven writing display is presented. This enables users to write and erase freely using a stylus connected to a low-power source. This system leverages an indium tin oxide (ITO) conductive film (10^3^–10^4^ S cm^−1^, optical transmittance greater than 80%) beneath the EIMC layer, where an applied voltage induces localized nanoparticle migration [30]. This movement results in high-contrast, rewritable text or drawings, potentially rendering the display helpful for interactive workspaces, or real-time brainstorming sessions.

Figure 3a1 illustrates the fundamental operating principle: a stylus, acting as an electrode, modulates the electric field at the point of contact, selectively redistributing electrophoretic NPs within the microcapsules to inscribe a clear emoji (figure 3a2). This effect produces visible writing with high resolution and short response times. Figure 3b demonstrates this mechanism in practice, where numbers (1, 2, 3, 4, 5) are visibly inscribed, confirming the responsiveness and functionality of our system, comparable to other commercial e-ink technologies. Figure 3c showcases other examples of freehand writing into well-defined shapes (e.g. arrow). These, defined by tape stencils, can be selectively toggled on/off via controlled electrical stimulation. This system has potential for customizable signage, yielding crisp, high-contrast patterns without blurring or smudging. Additionally, figure 3d displays a rudimentary matrix display, where a 3 × 3 grid of individually addressable cells was created using tape to partition the microcapsule layer. This demonstrated the feasibility of electrophoretic microcapsules for low-power digital signage, clocks or notification panels, where each electrophoretic cell can independently stream a different set of information. While the current system is a proof of concept, it highlights the potential for scalable, energy-efficient matrix displays, pending further development of addressing circuitry and control mechanisms.

To quantitatively assess the display performance, the drawing precision of the prototype was analysed. The theoretical minimum feature size of the display is approximately 25–50 µm (representing 1–2 microcapsule widths). In practice, the line resolution is dictated by the stylus geometry (size of electrode) and electric field fringing. The drawn line with a standard multimeter electrode is recorded and measured in imageJ, and the drawn line with 6033 ± 705 µm width was achieved. Edge sharpness was quantified via optical greyscale profiling, yielding a robust contrast ratio of 1.94 between the actuated regions and background. Furthermore, to validate the ‘flexible writing’ capability and mechanical durability, the EIMC layer was subjected to cyclic write–erase testing. After 100 continuous cycles driven at 20 V, the display exhibited no visible capsule rupture, maintaining a contrast ratio of 1.58, confirming the reversibility and mechanical resilience of the cross-linked coacervate shell under repeated electromechanical stress.

### 2.4. Mechanical performance of e-ink microcapsules

**Figure 4.**
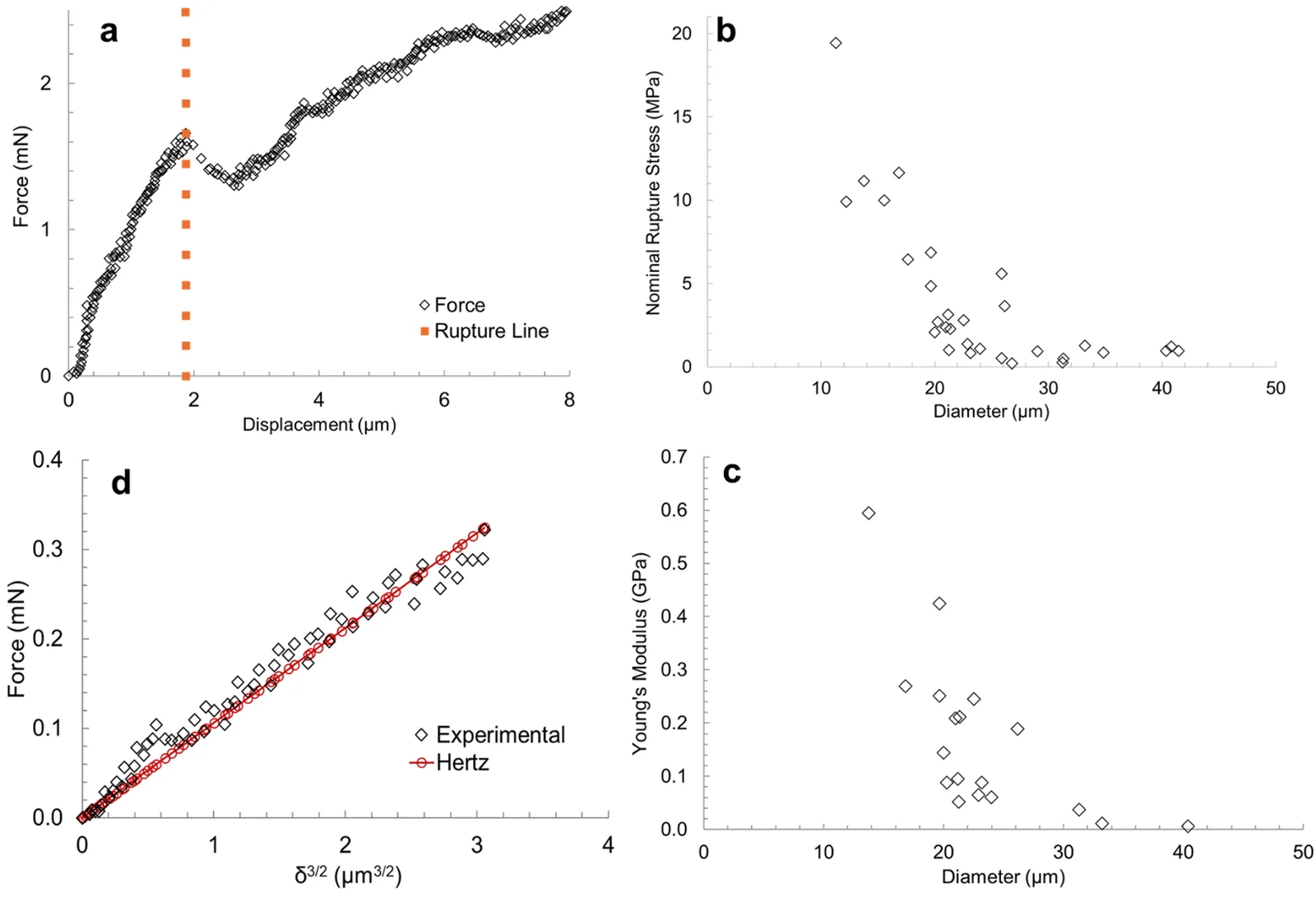
(a) Force–displacement curve of gum acacia–gelatin microcapsules under compression, showing a rupture event followed by a residual mechanical response; (b) nominal rupture stress as a function of diameter, indicating higher stress in smaller capsules; (c) apparent Young's modulus versus diameter; (d) typical experimental force–displacement curve fitted to the Hertz model (*R*^2^ ∼ 0.963).

The mechanical performance of gum acacia–gelatin EIMCs encapsulating electrophoretically active gentian violet dye (GVD) with surface-modified TiO_2_ NPs was assessed through a micromanipulation technique [31,32]. The average *D*_nb,m_ = 23.2 ± 1.5 µm (*n* = 30) obtained by micromanipulation was in excellent agreement with *D*_nb,J_ (22.4 ± 0.4 µm). The typical force–displacement curve (figure 4a) reveals a progressive force increase until rupture, occurring at an average displacement of 1.9 ± 0.3 µm, and marked by a sharp force drop (see the vertical orange dashed line). This highlights that the microcapsules can withstand significant compression before fracturing (average rupture force 1.14 ± 0.13 mN), which is a crucial property for durable applications, especially in electrophoretic displays/cells.

Figure 4b illustrates the relationship between the nominal rupture stress of EIMCs and their diameter, which appears to be inversely proportional to each other. Similar behaviours were described by Hu *et al.* [33]. The mean rupture stress was found to be 3.9 ± 0.8 MPa, significantly higher than commercial, microplastic-based melamine-formaldehyde microcapsules, used in a number of consumer products (1.2–1.8 MPa) [34]. This enhancement in mechanical strength can be attributed to the cross-linked shell structure and the reinforcing effect of TiO_2_ NPs. It cannot be excluded that some NPs may have got integrated within the coacervated shell matrix during encapsulation [26,35].

Compared with other microcapsules reported in the literature, such as those with polydopamine (PDA)-coated calcium carbonate (2.2 ± 0.3 MPa) shells, these new EIMCs exhibited superior rupture stress, rendering them probably suitable for high-stability electronic ink applications where mechanical strength is pivotal.

The Young's modulus of the whole EIMCs, which is a measure of their apparent stiffness [36], was found to be 0.17 ± 0.03 GPa (figure 4c), determined by the Hertz model [31]. Interestingly, this value suggests that EIMCs are relatively rigid prior to rupturing, and then plastically deforming, under compression (deformation at rupture approx. 18% ± 2%). Furthermore, there appeared to be a tentative correlation between the elastic modulus of EIMCs and their diameter. This probably indicates less stiff microcapsules at larger diameters, which is possibly owing to their ratio of shell thickness to diameter becoming smaller. Similar considerations were reported elsewhere [17]. Typical experimental force–displacement data fitted by the classical Hertz model is displayed in figure 4d, featuring a coefficient of determination (*R*^2^) greater than 0.96.

Overall, the analysis of the mechanical properties of EIMCs suggests that they may be well-suited for electrophoretically assisted applications, especially for on-skin digital interfaces, where dynamic NP movement within an electric field and resilience against mechanical fatigue are required. The key mechanical parameters are summarized in table 1.

**Table 1.** Mechanical properties of gum acacia-gelatin EIMCs for assessing the robustness of EIMCs.

	mean ± s.e.
number-based diameter *D*_nbm_ (µm)	23.2 ± 1.5
displacement at rupture (µm)	4.6 ± 0.8
rupture force (mN)	1.14 ± 0.13
deformation at rupture (%)	17.9 ± 2.3
nominal rupture stress (MPa)	3.9 ± 0.8
rupture tension (µN µm^−1^)	55.6 ± 8.5
apparent Hertzian Young's modulus, *E_H_* (GPa)	0.17 ± 0.03

### 2.5. Fourier transform infrared and surface charge of nanoparticles

**Figure 5.**
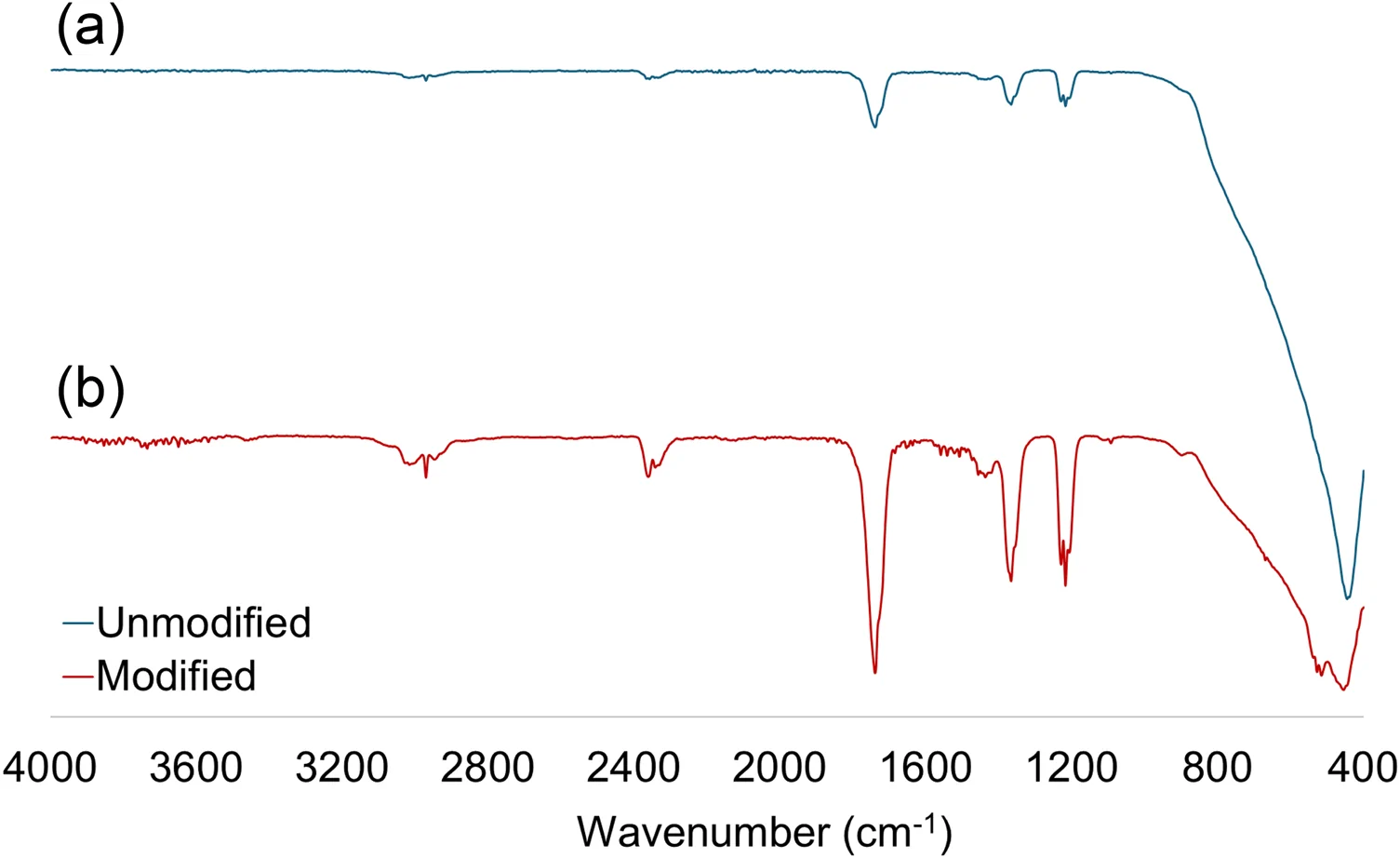
FT-IR spectra of (a) unmodified and (b) surface-modified TiO_2_ NPs. New peaks at 1720 cm^−1^ (C═O stretching) and 1150–1250 cm^−1^ (C–O–C stretching) confirm polymer grafting, whereas reduced O–H stretching (approx. 3200–3500 cm^−1^) indicates decreased surface hydroxyl groups.

Fourier transform infrared (FT-IR) analysis (figure 5) revealed significant changes after the surface modification of TiO_2_ NPs with ethylene glycol dimethacrylate (EGDMA) and methyl methacrylate (MMA), indicating successful polymer grafting. The unmodified TiO_2_ spectrum (a) shows characteristic Ti–O–Ti stretching vibrations and Ti–O bonds approximately 500–800 cm^−1^, typical of anatase-phase TiO_2_, with its largest frequency absorption at 456 cm^−1^ [37]. Additional bands at 1217 cm^−1^ and approximately 1700 cm^−1^ are ascribed to the deformation vibration of the Ti–OH stretching mode [38].

After modification (b), the emergence of stronger peaks in the 1720 cm^−1^ region corresponds to C═O stretching vibrations from ester groups (─COOCH_3_). This probably suggests the presence of the co-polymer on the NP surface, resulting from the reaction of MMA and EGDMA. The weak oscillating absorption region near 3200–3500 cm^−1^ tentatively suggests a reduction in surface hydroxyl groups owing to their ratio co-polymer coverage. Additionally, peaks at approximately 900, 1100 and 1250 cm^−1^ correspond to C–O–C asymmetric stretching, further supporting polymerization. The presence of methacrylic acid (MAA) introduces carboxyl (─COOH) functionalities, possibly interacting with TiO_2_ via hydrogen bonding or coordination, enhancing stability.

Furthermore, surface-modified TiO_2_ NPs exhibited a more negative surface electrokinetic potential (SEKP) of −49.3 ± 0.6 mV compared with non-modified (−23.6 ± 0.9 mV) TiO_2_ NPs (supplementary material, S5). The higher the absolute SEKP, the higher the charge carried by the NPs, making them more stable (electrosteric repulsion), preventing agglomeration, thereby ensuring better performance of the electrophoretic medium [35,39]. Overall, the spectral shifts, coupled with the increased zeta potential, indicate successful surface modification through polymerization, improving the dispersion and hydrophobicity of TiO_2_ NPs.

### 2.6. Cytotoxicity and sensitization assays

**Figure 6.**
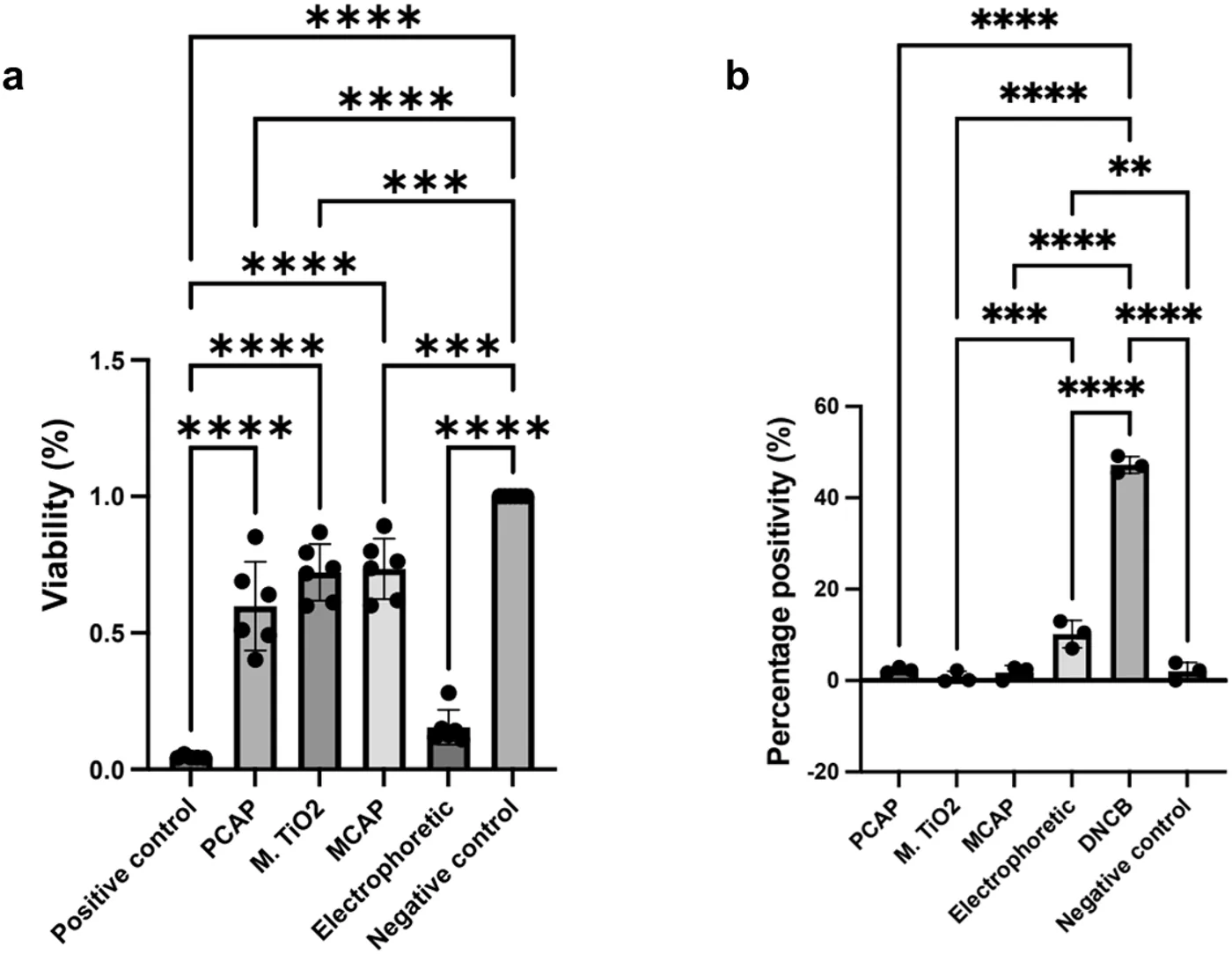
(a) Cell viability of different test groups compared with positive (SDS) and negative (keratinocyte culture medium) controls, including blank microcapsules (PCAP), surface-modified TiO_2_ NPs (M.TiO_2_), e-ink microcapsules (MCAP) and the absolute electrophoretic medium; (b) CD54 expression of different test groups at 100 µg ml^−1^, including DNCB (known sensitizer acting as a positive control), phosphate buffered saline (PBS) as a negative control and dissolved gelatin–gum acacia biopolymers (unreacted shell precursors, PCAP). Results are expressed as mean ± standard error. Error bars may be smaller than the symbol size. (*n* = 3, ****p < 0.0001, ***p < 0.001, **p < 0.01, *p < 0.05, ns *p* ≥ 0.05.) Ordinary one-way ANOVA with Tukey's multiple comparisons test.

The cytotoxicity assay results are shown in figure 6a, where the relative viability of the testing groups is defined as the ratio of neutral red fluorescence intensity of each testing group to the negative control (keratinocytes cultured with medium only). Therefore, the negative control exhibited near 100% viability, confirming baseline cell growth, whereas the positive control (cytotoxic agent) showed minimal viability, validating the assay.

Surface-modified TiO_2_ (M-TiO_2_) and microcapsules (EIMC) maintained relatively high viabilities (approx. 70%), indicating low cytotoxicity. Empty microcapsules (PCAP) exhibited slightly lower viability (approx. 58%). In contrast, the absolute electrophoretic medium may cause severe cytotoxicity, significantly reducing cell viability to approximately 15%. However, in practical formulations, it is applied only in very small amounts, which mitigates its overall impact. Future studies should therefore focus on engineering novel, highly biocompatible electrophoretic media. Overall, the viability profile of the formulation is balanced by the favourable performance of surface-modified TiO_2_ and microcapsules, both of which maintain relatively high cell viability.

Figure 6b presents the CD54 expression levels across different groups. The experiment was performed at 100 µg ml^−1^. The negative control exhibited a low baseline CD54 expression (approx. 5%), while the positive control (2,4-dinitrochlorobenzene (DNCB), a known sensitizer) induces a strong upregulation (approx. 32%), validating the assay. Among the tested materials, only the absolute electrophoretic suspension caused a relative increase in CD54 expression (approx. 12%), approximately twofold higher than the negative control, suggesting potential sensitization activity, as expected. In contrast, electronic ink microcapsules (EIMC 1.7%) are relatively safe to cells *in vitro*, as they prevent encapsulated electrophoretic suspension to be released into the surrounding environments. Surface-modified TiO_2_ (M-TiO_2_, 2.7%), and blank microcapsules (PCAP ∼ 3%) exhibited minimal impact, with CD54 levels close to baseline. These findings indicate that only the absolute electrophoretic suspension may trigger an immune activation response, and should be thus prevented from contacting skin directly in potential applications, whereas the other samples showed negligible sensitization potential since its amount in practical formulations is minimal.

Interestingly, both assays indicated that the formulated EIMCs showed limited cytotoxicity and sensitization potential. However, there are some aspects worth exploring. It cannot be excluded that surface-modified TiO_2_ nanoparticles, which carry negative charges, may aggregate in solution (e.g. phosphate buffered saline; PBS) owing to its higher ionic strength compared with deionized water [40]. Such aggregation could change the effective surface area, allowing cells to grow more readily and appear more confluent. In addition, the photocatalytic properties of surface-modified TiO_2_ NPs, in an aggregated status, may help mitigate cytotoxicity [41].

## 3. Discussion

This study aimed to evaluate whether microencapsulation could mitigate the potential biological hazards associated with high-dielectric electrophoretic solvents and nanoparticle-containing display inks. We hypothesized that microencapsulation would reduce effective solvent bioavailability, attenuating cytotoxicity and sensitization responses, while preserving electrophoretic performance for potential skin-contact applications. The present findings support this hypothesis, providing broader insight into the design of next-generation smart materials for electronic and biomedical applications.

### 3.1. Comparative analysis with existing technologies

To contextualize the advancement of this formulation, table 2 benchmarks the proposed EIMCs against established commercial products and prior literature. Commercial electrophoretic displays, such as the E Ink® Vizplex™ and Mobius™ architectures, rely on rigid plastic-based microcapsules including melamine-formaldehyde or urea-formaldehyde to contain their dielectric solvents. While these products achieve rapid switching speeds (approx. 0.2 s), they suffer from relatively low nominal rupture stress (1.2–1.8 MPa) and cytotoxicity issues. Conversely, previous attempts at biopolymer encapsulation often sacrifice electro-optical performance and lack rigorous tissue exposure validation. As demonstrated in table 2, our specific cross-linking strategy bridges this gap by quantitatively analysing the properties of biopolymer e-ink and its biosafety.

**Table 2.** Comparison of properties of different E-ink types.

characteristic	commercial standard [3, 7, 34]	prior academic (synthetic) [12, 13]	prior academic (biopolymer) [11, 18]	this work
**shell material**	melamine-formaldehyde	polyurea/PMMA	gelatin/gum acacia	gelatin/gum acacia
**core solvents**	halocarbon oils	Isopar/PCE/Aliphatic oils	variable (often mineral/vegetable oils)	HS-PCE
**fabrication process**	in-situ polymerization, electrospray	interfacial polymerization	complex coacervation	complex coacervation
**switching speed**	∼0.1–0.5 s	∼0.5–1.0 s	>1.0 s	∼0.5 s
**nominal rupture stress**	1.2–1.8 MPa	variable (brittle)	–	3.9 ± 0.9 MPa
**biocompatibility/sensitization**	potential cytotoxic	poor (uses toxic monomers)	assumed (rarely quantified)	quantified high

### 3.2. Electrophoretic performance and biocompatibility

Striking the right balance between dielectric performance and biological safety is a key challenge in wearable electrophoretic display design.

Electrophoretically active solvents, such as perchloroethylene (PCE), or blends thereof, with a low dielectric constant (ε_r_ < 5) are essential for high performance, enabling rapid particle motion, strong colour contrast and short response times [11]. However, they often exhibit poor biocompatibility, causing cellular membrane disruption and skin irritation, when contacting body tissues [42]. To this end, our encapsulated formulation provides a pathway to reconcile these challenging demands.

Interestingly, our findings suggest that it may be possible to retain high-performance electro-optical properties while reducing the toxicological risk. This was indeed achieved by segregating the internal electrophoretic medium from direct contact with epidermis, which yielded satisfactory biocompatibility and low sensitization.

This is particularly relevant for materials intended for on-skin applications, where electrophoretic displays must interface safely with the epidermis over prolonged periods [11,19].

Although our study did not directly quantify the permeability across the microcapsule shell, the consistently low sensitization implies that the solvent transfer is minimized, therefore yielding reduced toxicity [19,35,36].

These observations align with literature on microencapsulation by complex coacervation, which has demonstrated a substantial reduction in the permeability of actives through the shells, compared with unencapsulated actives [16,43]. Moreover, fragrance microcapsules using different shell chemistries also showed reduced active leakage, even for amphiphilic and/or highly volatile materials [44]. Overall, our microencapsulated formulation acts as a mass transfer resistance, segregating the solvent, protecting cells from direct solvent exposure, and reducing nanoparticle–cell interactions (minimized immune response).

### 3.3. Methodological considerations

The use of both THP-1 cells with CD54 activation markers and the neutral red uptake assay provided complementary insights into the cytotoxicity and immunotoxicity of our encapsulated formulation. THP-1 cells remain a widely used and OECD-endorsed model for predicting skin sensitization [45]. However, they represent only a simplified immune system and cannot reproduce the full range of immune interactions that occur in living tissue. Likewise, the neutral red uptake assay provides a reliable measure of overall cell viability, but does not detect more subtle, non-lethal cellular effects such as oxidative stress or early membrane damage. Future work may expand these findings by incorporating assays for inflammatory cytokines, oxidative stress markers, computational diffusion modelling and shell integrity testing under relevant mechanical loads.

### 3.4. Mechanical performance for biomedical translation

Beyond cytotoxicity, the translational relevance of encapsulated materials critically depends on their mechanical stability [32]. For skin-integrated systems, particularly soft, stretchable or deformable devices, microcapsules are subjected to mechanical compression, shear, bending and abrasion during normal use. In this context, the microcapsules are sandwiched within the device architecture and not directly exposed to the external environment; therefore, their mechanical stability should be considered under transmitted stresses (micromanipulation of single microcapsules) rather than direct surface wear of bulk microcapsule layers.

The mechanical performance of the microcapsules developed in this work is satisfactory and outperforms that of many single-use microcapsules currently employed in industrial applications [34]. Enhanced mechanical strength is especially important for small-scale displays for biomedical applications, where microcapsules are expected to function over extended periods in anatomically dynamic regions such as the knees or shoulders, which experience substantial mechanical stress. In the meantime, the biocompatible assays require EIMCs to be suspended in aqueous PBS, subjected to prolonged UV irradiation (greater than 30 minutes), and incubated at 37°C under extreme humidity (92% RH) for at least 24 hours. These conditions perfectly mimic the thermal and hydrolytic stressors of epidermal sweat exposure, thereby proving its environmental robustness. While extreme industrial environmental cycling (e.g. −10°C to +50°C) remains the focus of future device-level packaging work, the fundamental structural integrity of the bare EIMC formulation under physiological heat and saline exposure is empirically validated.

Although beyond the scope of this study, future investigations should assess long-term shelf stability and resistance to degradation to further validate the formulation for a broader range of healthcare applications, including dermatological uses. Potential examples include the encapsulation of electrophoretically active, skin-mimicking pigments for vitiligo camouflage, adaptive skin-tone materials and formulations for topical depigmentation or alopecia therapies. These applications require predictable mechanical behaviour in addition to an acceptable toxicological profile.

### 3.5. Regulatory considerations and translational relevance

Encapsulation intended for biomedical applications must satisfy relevant regulatory criteria to ensure safety at the point of use.

Gelatin and gum Arabic already hold regulatory acceptance in food, pharmaceutical and cosmetic products, positioning them within ISO 10993 and cosmetic safety frameworks [46]. Although the internal phase of our formulation is not inherently biocompatible, its biological impact can be dramatically mitigated through effective shell protection, as evidenced by the immunotoxicity assays.

Together, these results support a credible translational pathway in which materials development, toxicological assessment and regulatory considerations progress in parallel. While additional studies are required, particularly to evaluate long-term exposure and shelf-life, the current data indicate that microencapsulation provides a promising platform for relatively safe, high-performance electrophoretic materials in both electronic and healthcare-oriented applications.

### 3.6. Interfacial physics and molecular interactions

The functional integration of the proposed EIMCs into wearable architectures relies on the interfacial mechanics between the microcapsules, the binder matrix and the target substrate.

#### 3.6.1. Adhesion mechanisms and colloidal stability

The adhesion between microcapsules and substrate is driven by the binder (mixture of polyvinyl acetate (PVA), poly(vinyl pyrrolidone) (PVP) and gelatin). At the molecular level, the chemical groups in PVA and PVP form strong hydrogen bonds and dipole interactions with both the microcapsule shells and the underlying surface [47]. This adhesive mixture also forms a flexible, shock-absorbing network that dissipates mechanical stress when the material is bent. This prevents the microcapsule layer from peeling or cracking, allowing the display to dynamically conform to textured, skin-like surfaces [8].

#### 3.6.2. Electrical driving mechanism

During electrophoretic activation, the gelatin/gum acacia shell serves as a biological insulating interface that electrically screens the TiO_2_ nanoparticles from direct electrical contact with the external electrodes. This insulation suppresses current flow, reducing the risk of electrochemical degradation of the organic solvent inside the microcapsule [7]. Owing to its high dielectric strength (approx. 19 kV mm^−1^), the shell transmits the applied electric field without electrical breakdown, while allowing Coulombic forces to actively drive the surface-modified TiO_2_ nanoparticles [48]. As a result, the particles remain responsive to the electric field without becoming permanently trapped at the electrode interface [3].

#### 3.6.3. Molecular basis of biocompatibility

The microcapsule shell forms the biological interface and is the primary determinant of biocompatibility. While the electronic ink contains potential cytotoxic solvents, the cross-linking process consumes reactive primary amines to form covalent cross-links, yielding a dense, chemically stable shell. This structure minimizes interactions between the shell material, the encapsulated chemicals and environmental biomolecules (e.g. enzymes) [49]. As a result, this barrier acts not only as a protective barrier that confines the internal contents, but also as an interface that limits the adsorption of proteins, antibodies and enzymes onto the microcapsule surface, thereby enhancing safety during contact with epidermal tissues [50].

## 4. Conclusion

In conclusion, this work resolves the electro-toxicological bottleneck of wearable electrophoretic displays. By repurposing complex coacervation as an interfacial engineering tool, we developed cross-linked gelatin-gum acacia microcapsules that act as robust shields to prevent cytotoxicity. This specific architecture achieves high mechanical resilience (nominal rupture stress of approx. 3.9 MPa) and rapid switching (approx. 0.5 s) while empirically mitigating the cytotoxicity and CD54 sensitization of high-performance dielectric solvents, providing a foundational platform for cytocompatible, flexible epidermal bioelectronics.

## 5. Material and methods

### 5.1. Materials (microencapsulation)

All chemicals used in this study were sourced from Sigma (Gillingham, Dorset, UK), unless otherwise specified. Specifically, anatase titanium dioxide nanoparticles (TiO_2_ NPs; purity 99.8%; CAS Number: 1317-70-0; 30 nm; approx. 3.8 g cm^−3^), a lightweight variant owing to its lower density compared to rutile (approx. 4.3 g cm^−3^), were employed. Bovine hide gelatin (type B; CAS Number: 9000-70-8) and gum acacia (CAS Number: 9000-01-5) served as shell precursors, while hexyl salicylate (CAS Number: 6259-76-3) and tetrachloroethylene (perchloroethylene (PCE), CAS Number: 127-18-4) were used as components of the liquid medium. Sorbitan trioleate (Span 85; CAS Number: 26266-58-0) functioned as a steric surfactant, while poly(vinyl pyrrolidone) (PVP) and ethylene glycol dimethacrylate (EGDMA) acted as surface modifiers and stabilizers. 1-Propanol (PrOH; CAS Number: 71-23-8) was used as a dilution solvent, and methyl methacrylate (MMA; CAS Number: 80-62-6) and methacrylic acid (MAA; CAS Number: 79-41-4) were used as the monomeric building blocks. Glacial acetic acid (CAS Number: 64-19-7) and sodium hydroxide (NaOH; CAS Number: 1310-73-2) were employed as pH adjusters. Gentian violet dye (GVD; CAS Number: 548-62-9; 0.5% working solution; ethanol less than 10% (v/v); APC Pure, Cheshire, UK) was incorporated as a dark pigment, while DL-glyceraldehyde (10%; CAS Number: 56-82-6) served as a cross-linking agent. Potassium bromide (KBr; CAS Number: 7758-02-3) was used as an infrared (IR)-transparent window material for characterization, and ethanol (CAS Number: 64-17-5) was employed as a cleansing agent. Azobisisobutyronitrile (AIBN; CAS Number: 78-67-1) functioned as an initiator. All chemicals were of analytical grade, stored in accordance with their respective Safety Data Sheet guidelines, and used without further purification. All solutions were prepared using Milli-Q water (18.2 MΩ cm^−1^ at 25°C).

### 5.2. Materials (bioassays)

Commercial normal human epidermal keratinocytes (NHEK) cells, isolated from the epidermis of adult skin from single/pooled donors, were purchased from PromoCell (Heidelberg, Germany) and used as the test model for cytotoxicity. Neutral red (CAS Number: 553-24-2) served as a dyeing indicator for evaluating cell viability. Phosphate buffered saline (PBS) was used for washing steps. Trypsin–EDTA (0.05% w/v trypsin, 0.02% w/v EDTA; CAS Number: 9002-07-7) facilitated cell detachment. Foetal bovine serum (FBS) was used as trypsin neutralizer. Sodium dodecyl sulphate (SDS; CAS Number: 151-21-3) was used as a positive control. Keratinocyte Growth Medium 2 (PromoCell, Product number C-39016, Lot number 513M060) was used for NHEK cell culture and cytotoxicity assay to provide essential nutrients and antimicrobial protection. RPMI1640 media (Gibco, catalogue number 11875093) supplemented with l-glutamine (CAS Number: 56-85-9), penicillin–streptomycin (Gibco, Penicillin G Sodium Salt; CAS Number: 69-57-8), Streptomycin sulphate CAS Number: 69-57-8 3810-74-0) was used in culturing THP-1 cells and sensitization assay. Zwitterionic Gibco HEPES (4-(2-hydroxyethyl)-1-piperazineethanesulfonic acid) was used to wash and prepare the NHEK cells for cytotoxicity assay. (Life Technologies, ThermoFisher Scientific, USA). Glacial acetic acid (CAS Number: 64-19-7) and 96% (v/v) ethanol (CAS Number: 64-17-5) were used for fixation. Lastly, 0.4% (w/v) Trypan blue in 0.9% (w/v) NaCl solution (CAS Number: 72-57-1) was employed to assess cell membrane integrity. All chemicals were obtained from Sigma–Aldrich (Gillingham, Dorset, UK), unless otherwise specified.

### 5.3. Surface modification of TiO_2_ nanoparticles

Pristine anatase TiO_2_ NPs (4.0 g) were suspended in a hydropropanolic solution of PVP (2.0 g; 0.2 l of PrOH) contained in a double-glazed jacketed vessel (capacity 0.5 l) thermostated at 45.0 ± 0.1°C (Dyneo DD300F, Julabo UK Ltd., Stamford, UK). The suspension was continuously stirred over 30 min using a mechanical overhead stirrer (IKA Eurostar 20, Germany) fitted with a six-blade Rushton impeller (diameter Ø 34 mm; blade dimension 15 × 7.5 mm).

**Figure 7.**
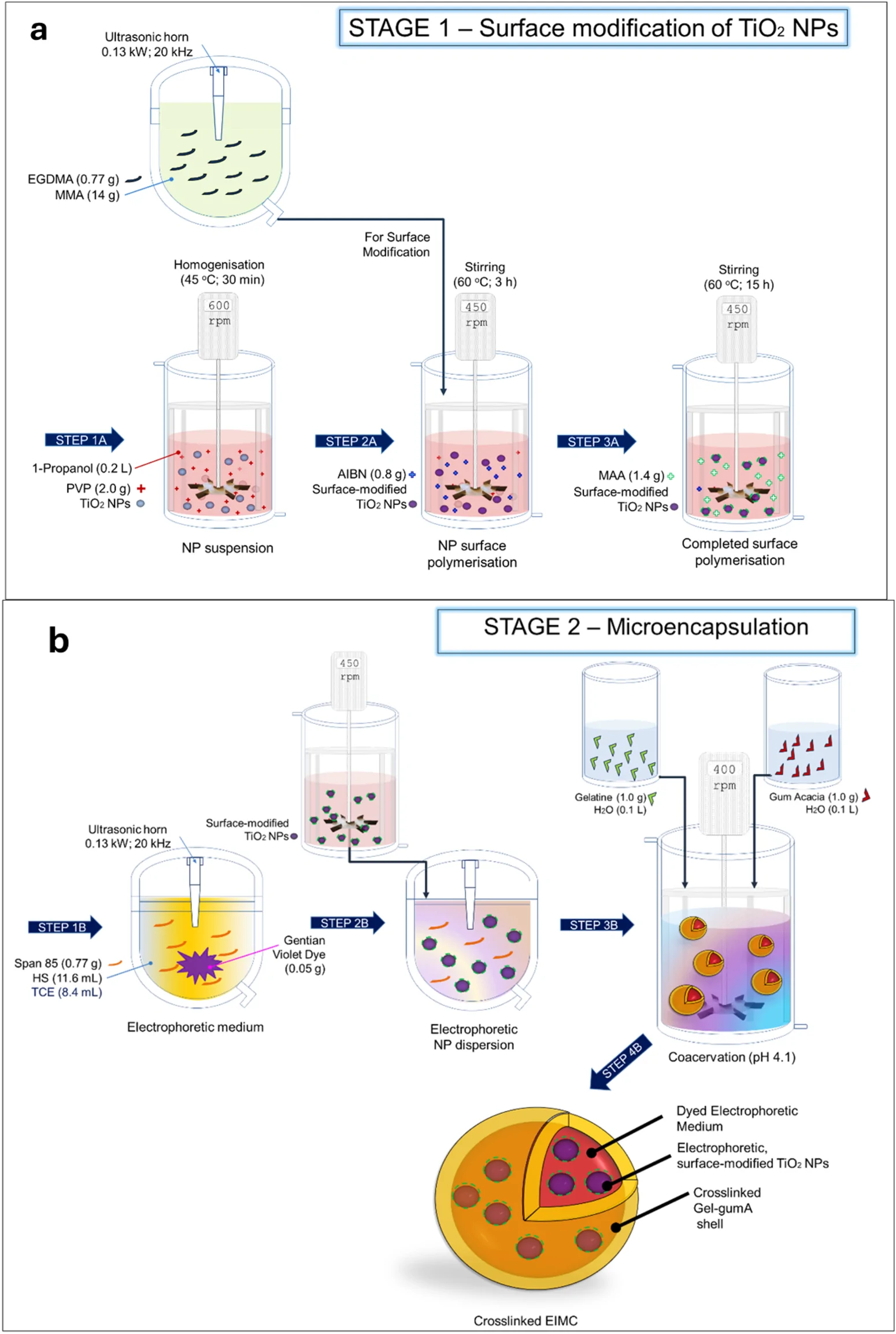
Experimental procedure for (a) surface modification of TiO_2_ NPs via polymerization of EGDMA and MMA and (b) fabrication of e-ink microcapsules, laden with GVD and TiO_2_ NPs, via complex coacervation of gum acacia and gelatin at pH 4.1.

Meanwhile, at room temperature (22.5 ± 1.0°C), MMA (14.0 g) and EGDMA (0.77 g) were combined in a beaker and sonicated for 10 min using an ultrasonic processor horn (operational parameters: 0.13 kW, 20 kHz; amplitude 60%; 2 : 1 (s) pulse sequence; probe diameter 20 mm; Vibracell, Sonics & Materials, Inc., Newtown, CT, USA). The homogenized monomer admixture was then introduced into the TiO_2_–PVP–hydropropanol solution and subjected to mechanical stirring (10 min; 450 r.p.m.). AIBN (0.8 g) was added to induce polymerization. The reaction proceeded at 60°C under continuous mechanical stirring (450 r.p.m.; 3 hours). During this stage, MMA and EGDMA formed a hydrophobic polymeric coating around the TiO_2_ NPs, reducing their relative density compared with uncoated TiO_2_. MAA (1.4 g), as the stabilizing co-monomer, was added slowly, introducing the carboxyl (─COOH) functional groups; surface modification was continued under unchanged processing conditions for approximately 15 hours. The incorporation of MAA imparted a strong surface charge to the NPs, thereby enhancing stability and mobility within the liquid medium. Following modification, the resulting admixture was allowed to settle to facilitate the separation of solid particles from the solvent. The particles were then thoroughly washed with Milli-Q water to remove any residual reactants. Figure 7a illustrates the procedure for surface modification.

### 5.4. Preparation of the coloured electrophoretic medium

The electrophoretic coloured liquid medium consisted of HS (11.6 ml) and PCE (8.4 ml), primed with Span85 (0.1 ml) as a surfactant. This mixture was ultrasonically homogenized (0.13 kW, 20 kHz; amplitude 60%; 2 : 1 (s) pulse sequence) for 5 min at room temperature (25°C). To enhance contrast, GVD (5 wt%) was incorporated yielding a darker colour. The resulting medium was ultrasonically homogenized again following the same condition as above, achieving a relatively fine dispersion. Subsequently, the surface-modified TiO_2_ NPs were introduced, and the admixture underwent an additional round of sonication (15 min). Supplementary material, figure S1 shows the admixture before and after sonication.

### 5.5. Microencapsulation

The microcapsule shell was formed by complex coacervation, with a biopolymer matrix of gum acacia (p*K*_a_ ∼ 2.2) and gelatin (p*K*_a_ ∼ 5). A 1 wt% gelatin solution was prepared by dissolving 1.0 g of gelatin (Gel) in 0.1 l of deionized water at 50°C under continuous stirring (700 r.p.m.). The previously prepared electrophoretic medium was added to the Gel solution to form the oil phase droplet (500 r.p.m.; 45 ± 0.1°C; pH 5.15). Separately, a 1 wt% gum acacia (gumA) solution was prepared under the same conditions. The two solutions were dropwise combined, and then stirred for an additional 1 hour while thermostated at 45 ± 0.1°C. The pH was gradually adjusted to 4.1, with aqueous acetic acid, at a controlled flow rate (0.25 ml min^−1^) through an infusion pump (Ultra 70-3007, Harvard Apparatus, MA, USA). The formation of microcapsule shell was observed under bright-field microscopy (Leica DM RBE microscope, Leica Microsystems GmbH, Dassel, Germany). Shell hardening was achieved by cross-linking, using aqueous glyceraldehyde (10 ml), while the solution was maintained at 5°C for approximately 14 hours (overnight) to allow complete shell reticulation. The temperature was then increased to 45°C for 3 hours to improve cross-linking efficiency and harden the shell. Figure 7b illustrates the experimental set-up for fabricating EIMCs.

### 5.6. Microcapsule post-processing

After encapsulation, the resulting microcapsules were filtered and thoroughly washed with deionized water to remove possibly unreacted components. During each wash, the microcapsules were centrifuged using a Sigma 4-16KHS centrifuge at 1000 r.p.m. for approximately 5 min. The wet microcapsules (slurry) were then dried in a drying cabinet at 40 ± 0.5°C for 24 hours (ε^3^ Genlab, GPE Scientific Ltd., UK) to evaporate residual moisture and solvents. For the biocompatibility tests, microcapsules were vacuum-sieved (filter paper: Whatman grade 1, pore size 11 µm, Product number WHA1001070; vacuum pump: VWR/ KNF PM 20405-86 Diaphragm pump, Protection class: IP20 Final pressure: 100 mbar), collected and temporarily stored in an airtight container prior to being analysed.

## Supplementary Material



## Data Availability

The data supporting the findings of this study are provided within the manuscript and its electronic supplementary material [51].

## Appendix

### A.1. Analytical techniques

#### (a) Structural and morphological properties

Structural and morphological properties of the microcapsules were analysed using both optical and scanning electron microscopy (SEM). Bright-field optical imaging was conducted with a Motic Pro 252 digital camera (Motic Deutschland GmbH, Wetzlar, Germany) and processed via Motic Images Plus 2.0 software. PL Fluotar 5×/0.12 and 10×/0.30 objective lenses were used for enhanced visualization. SEM was employed to assess surface structure and morphology (Hitachi TM3030 benchtop, Hitachi HighTech, Japan), operating at magnifications between 15× and 30 000×. The system featured high-resistivity silicon drift detectors (Bruker Quantax 70, Bruker Corporation, Billerica, MA) for elemental examination through energy-dispersive X-ray (EDX) spectroscopy. Imaging was performed under high vacuum conditions (less than 10^−3^ Pa) at an accelerating voltage of 15 kV, using a backscattered electron detection mode with a built-in four-segment detector. For sample preparation, approximately 0.1 ml of a 0.2 wt% microcapsule suspension was applied to an aluminium pin stub with adhesive carbon tape. To improve conductivity, an approximately 8 nm platinum coating was applied under vacuum (less than 10^−2^ Pa) via a Polaron Sputter Coater SC7640 (QuorumTech, Sussex, UK) before imaging.

#### (b) Particle sizing

The mean particle size was assessed using optical microscopy, with real-time imaging of EIMCs. The acquired SEM micrographs were processed and analysed using ImageJ freeware (Version 1.53t, National Institutes of Health, USA), for the quantification of number-based diameter (*D*_nb,J_; *n* = 200). More details are available in the supplementary material.

#### (c) Zeta potentiometry

The electrokinetic potential (EKP) was determined by zeta potentiometry (Zeta Sizer Ultra, Malvern Panalytical, Malvern, UK) at 25 ± 0.2°C to quantify the surface charge of TiO_2_ NPs. Measurements were conducted at the required pH (approx. 4.1); 0.1 M HCl_aq_ or NaOH_aq_ were employed as pH adjusters. Ultrapure deionized water (18.2 MΩ cm^−1^) with 1 mM NaCl was used as the background electrolyte. The TiO_2_ NP suspensions were analysed through folded capillary cells (DTS1070, Malvern, UK), which were thoroughly rinsed thrice with deionized water prior to testing. For objects (e.g. NPs, microcapsules) with a well-defined surface, the terminology EKP and zeta potential can be used interchangeably [16].

#### (d) Fourier transform infrared spectroscopy

Fourier-transform infrared (FT-IR) spectroscopy was employed to analyse the dried samples using a PerkinElmer FT-IR GX System, integrated with an attenuated total reflection (ATR) accessory (DuraSampleIR). Spectral data were collected over a wavenumber range of 4000 to 400 cm^−1^ at a resolution of 4.0 cm^−1^, with each spectrum averaged over 200 scans to enhance signal accuracy. For solid-state analysis, approximately 8 mg of the sample was uniformly blended with anhydrous potassium bromide (KBr) powder (0.5 g) to form an IR-transparent matrix. The mixture was compressed into pellets using a semiautomatic rotary tablet press (LFA Tablet Presses, UK), at 80 kN over 2 min, to improve infrared beam transmission. To prepare NPs and microcapsules for FT-IR analysis, a 15 ml suspension was first frozen at −21°C for 8 hours in pre-pierced polyethylene grip-seal bags (5 × 7 cm^2^). The frozen microcapsules were subsequently freeze-dried under vacuum (less than 0.04 kPa) at −53°C using a ScanVac CoolSafe Pro (DVS1000, Labogen, Denmark) until fully desiccated, yielding a free-flowing powder suitable for FT-IR assessment.

#### (e) Mechanical properties

The mechanical properties of individual microcapsules were assessed through a micromanipulation technique based on parallel plate compression [31]. Approximately 0.15 ml of a diluted microcapsule suspension was dispensed in two droplets onto a smooth glass slab (25 × 10 mm, thickness 3 mm) and left to air dry. The glass slab was then secured onto a metal stage beneath a borosilicate glass probe (1 mm diameter, flat-end tip approx. 80 µm wide) connected to a force transducer (GSC010, Transducer Techniques, USA) with a sensitivity of 8.674 mN V^−1^. The system was mounted on a three-dimensional micromanipulator equipped with a servomotor (DC Servocontroller, CONEX-C, USA) for precise movement, allowing the probe to descend at a controlled speed of 10 µm s^−1^. A high-magnification side-view camera (MVL12X12Z - 12X Zoom Lens with 12 mm Fine Focus, Thorlabs Inc, USA) recorded compression while force–displacement data were collected. Thirty microcapsules were randomly selected for compression to ensure statistical relevance. System compliance was calibrated thrice prior to compression, and the average value was used to correct displacement. The Young's modulus (*E_H_*) of EIMCs microcapsules was evaluated based on the classical Hertz model (equation A 1),

(A 1) $$\;E_{H}=\;\varphi \;F_{exp\;}\delta^{-3/2}R^{-1/2},$$

where *F_exp_* denotes the measured compression force, *δ* is the displacement at approximately 10% strain, *R* is the initial microcapsule radius and *ϕ* = 9/16 is a geometric coefficient accounting for both spherical shape and Poisson ratio (ν = 0.5) for incompressible rubber-like matter [36].

#### (f) Design and operation of prototype electrophoretic displays (PECDs)

To evaluate their deposition and performance, EIMCs were finely dispersed onto an indium tin oxide (ITO)-coated polyethylene terephthalate (PET) film (In_2_O_3_·(SnO_2_)_x_-PET; size 50 **×** 50 mm; resistivity 60 Ω sq^−1^; Merck, Darmstadt, Germany), which is a transparent conductive material for electrochromic displays, to facilitate charge injection. Specifically, EIMCs were suspended in an aqueous solution containing 2% gelatin, 5% PVP and 5% PVA (w/w), which provided structural support and stability. The EIMC paste was contained in a test tube and homogenized using a vortex mixer (2800 r.p.m., 0.6 A; Mini vortex mixer, Fisher Scientific, Leicestershire, UK), resulting in a relatively viscous medium. Coating of ITO PET films was performed by manually spreading the paste through a 200-mesh screen using cotton buds or a scalpel to ensure homogeneous distribution of EIMCs on the ITO surface. The microcapsule layer was activated by applying a 20 V DC potential through an electrode pair (Tenma, benchtop digital control DC power supply, model 72-10480, 3A, Japan), demonstrating electrophoretic response. Additionally, three prototype displays were developed to illustrate the applicability of EIMCs, operating at low voltage.

#### (g) E-ink microcapsule cytotoxicity

The cytotoxicity of e-ink is assessed by determining its effect on NHEK. NHEK cells were cultured in keratinocyte growth media as per the manufacturer's instructions. Cells were maintained at 80% confluency and used for cytotoxicity assay using neutral red dye (NRD) uptake assay as previously reported [52].

NHEK cells were seeded at 10 000 cells/well in a 96-well flat-bottom culture plate and allowed to attach under normal culture conditions (37°C, 5% (v/v) CO_2_, 92% relative humidity) for 24 hours. Then media was exchanged with 100 µl of testing suspensions in triplicate. SDS (at a concentration of 100 µg ml^−1^) was added as a positive control as described elsewhere [53,54]. Test samples including (i) surface-modified TiO_2_ NPs, (M-TiO_2_) (ii) e-ink microcapsules (EIMCs), (iii) blank microcapsules (PCAP, i.e. free from TiO_2_ and organic dye) and (iv) the electrophoretic suspension were added at a concentration of 1000 µg ml^−1^ for an initial assessment of toxicity.

For cell seeding, NHEK cells were rinsed with HEPES first, then incubated in 5 ml trypsin–ethylenediaminetetraacetic acid (EDTA) solution (0.05% w/v trypsin, 0.02% w/v EDTA) to detach the cells from T75 flask. After incubation trypsin was neutralized with 5% (v/v) FBS and centrifuged at 200*g* for 5 min. The cells were then resuspended in complete culture medium. Cells were counted using an automated cell counter (Countess II FL, Invitrogen™, ThermoFisher Scientific, USA), to check for cell viability (at least 90% recommended).

For cell treatment, test solutions were prepared fresh and diluted with PBS. The dielectric suspension and SDS were sterilized using a 0.22 µm filter, whereas EIMCs were UV-sterilized (Crossbeam UV light integrated in Thermo Safe 2020 Class II Biological Safety Cabinet, Fisher Scientific, UK) owing to their size (EIMC size ≫ 0.22 µm). All samples were placed 0.4 m away from the UV light (254 nm) and allowed for sterilization over 30 min. Surface-modified TiO_2_ NPs were UV-sterilized, since they may aggregate after surface modification. Subsequently, the samples were placed into designated wells, and duplicates were performed. Controls included blank wells, untreated cells and SDS (supplementary material, figures S1 and S1.1). Plates were incubated with the testing conditions for 24 h under standard culture conditions.

For NRD uptake assessment, the medium was removed from the wells and centrifuged (600*g*, 10 min) to remove precipitated dye crystals. Testing samples were separated from the cells and were washed with PBS at least three times.

An NRD stock solution (4 mg ml^−1^) was prepared by dissolving 40 mg NRD in 10 ml PBS (Ca^2+^- and Mg^2+^-free) and stored for up to two months at room temperature (20–30°C) protected from light. The NRD working solution (40 µg ml^−1^) was freshly prepared by diluting the stock solution 1 : 100 in culture medium and incubated overnight at the same temperature as the cultured cells. The NRD destain solution was prepared as a mixture of 50% ethanol (96%), 49% deionized water and 1% glacial acetic acid.

Subsequently, 100 µl of neutral red working medium was added to each well of plate and incubated for 2 hours at same culture conditions. After incubation with neutral red, wells were washed again with PBS, followed by the addition of 150 µl destain solution. Plates were shaken for at least 10 min to extract the dye, and optical density (OD) was measured at 540 nm using a microplate reader (Bio-Rad Benchmark, Hercules, USA). Viability was calculated by dividing the absorbance of treated cells by that of untreated cells to measure viability (%).

#### (h) E-ink microcapsule biocompatibility

The skin sensitization potential of EIMCs is determined using the human cell lines activation test (h-CLAT) by determining the expression of CD54 in THP-1 cells following exposure to the e-ink via flow cytometry, compared with positive and negative controls. This is a commonly used test as THP-1 cells overexpress CD54 upon exposure to sensitizers. Specifically, THP-1 cells were seeded at a density of 1 × 10^6^ cells ml^−1^ in 48-well plates, with 1000 µl per well. After 24 hours, cells were exposed to 100 µg ml^−1^ of the testing sample for an additional 24 hours at 37°C in a 5% CO_2_ environment. The tests were performed in triplicate.

Following incubation, the cells were prepared for flow cytometry. First, the cells were centrifuged (100*g*, 6 min), washed twice with PBS containing 1 wt% BSA (bovine serum albumin), and resuspended in a 96-well round-bottom plate at 1 × 10^5^ cells per well. The cells were then stained using a panel prepared in PBS + 1% BSA, with CD54-APC antibodies at 1 : 50 dilution, alongside corresponding IgG1-APC isotype controls at 4°C for 30 min. Following washing, the expression of cell surface CD54 was analysed using flow cytometry (Miltenyi MACSQuant Analyzer 16 Flow Cytometer) and analysed using flowjo software V (Version 10). Dead cells were excluded using PE-igG1 isotype controls (Miltneyi Biotec, UK).

In the sensitization assay, 2,4-dinitrochlorobenzene (DNCB) was used as a model sensitizer (positive control), and the tested samples (supplementary material, figures S2 and S2.1) were the electronic microcapsules (EIMC), surface-modified TiO_2_ (M-TiO_2_) and blank microcapsules (PCAP).
