# Supplementary material for: Bio-interfacial engineering of electrophoretic coacervate microcapsules for cytocompatible on-skin displays
Source: J R Soc Interface. 2026 Sep 9;23(242):20260105. doi: 10.1098/rsif.2026.0105 (PMC13561038; doi:10.1098/rsif.2026.0105)
Supplement: Supplementary file 1 [file rsif.2026.0105.zip › rsif.2026.0105/rsif20260105_si_001.docx]

**Title**: Engineering biocompatible E-Ink microsystems for flexible, wearable, on-skin displays with potential diagnostic applications

**Authors:** Wenda Zhao^1^**^†^**, Daniele Baiocco^2,3,4†^, Asme Boussahel^5^, Liam M. Grover^2,4^, Zhibing Zhang^2^, Anne Roudaut^1*^

**^1^** Department of Computer Science, University of Bristol, BS8 1QU, Bristol, UK

**^2^** School of Chemical Engineering, University of Birmingham, Birmingham, B15 2TT, UK

**^3^** Department of Chemical Engineering, School of Aeronautical, Automotive, Chemical and Materials Engineering (AACME), Loughborough University, LE11 3TT, UK

**^4^** Healthcare Technologies Institute, Institute of Translational Medicine, Queen Elizabeth Hospital, Birmingham, B15 2TH, UK

**^5^** Biomedical Science Building, University of Bristol, BS8 1TD, Bristol, UK

[*Anne.Roudaut@bristol.ac.uk](mailto:*Anne.Roudaut@bristol.ac.uk)

†These authors contributed equally to this work.

**SUPPORTING INFORMATION**

The supporting information demonstrates ultrasonic treatment results of the TiO_2_ NP suspension, Biocompatibility assays setup, and properties of TiO_2_ and EIMCs.

**Figure S1.**

Visual comparison of the TiO_2_ NP suspension before and after sonication, illustrating the dispersion enhancement achieved through ultrasonic treatment: (A) shows the initial biphasic state (top: sorbitan trioleate, hexyl salicylate and tetrachloroethylene (~1.25 g/cm^3^); bottom: gentian violet dyed NPs (~4 g/cm^3^), after surface modification, while (B) highlights the uniformly dispersed nanoparticles after 15 minutes of targeted sonication.

**Figure S2.**

Lab plate setup for the Neutral Red Uptake (NRU) assay. Controls included blank wells (C1, C12), untreated cells (C11), and SDS as a positive control (C2). Testing groups were electrophoretic suspension (C3, C4), modified TiO_2_ (C5, C6), E-ink microcapsules (C7, C8), and pure microcapsules (C9, C10).

**Figure S2.1**.

A

B

C

D

E

F

G

H

2

1

3

4

5

6

7

8

9

10

11

12

Detailed diagram of plate setup for the Neutral Red Uptake (NRU) assay. Controls included blank wells (C1, C12), untreated cells (C11), and SDS as a positive control (C2). Testing groups were electrophoretic suspension (C3, C4), modified TiO_2_ (C5, C6), E-ink microcapsules (C7, C8), and pure microcapsules (C9, C10).

**Figure S3.**

Flow cytometry lab setup for the CD54-APC antibody-based sensitisation assay. 2,4-dinitrochlorobenzene (DNCB) was used as a positive control. Testing groups included electronic microcapsules (EIMC; repeated (r)), surface-modified TiO₂ (M-TiO₂), and blank microcapsules (PCAP).

**Figure S3.1.**

**8**

**7**

**6**

**4**

**5**

**3**

**2**

**1**

**A**

**B**

**C**

**D**

**E**

**F**

Detailed diagram for the CD54-APC antibody-based sensitisation assay. Control groups include untreated cells C2, positive control 2,4-dinitrochlorobenzene (DNCB, C3). Testing groups included electrophoretic suspension (C4), electronic microcapsules (EIMC, C5), surface-modified TiO₂ (M-TiO₂, C6), and blank microcapsules (PCAP, C7).

**Table S1.**

| # | ImageJ  diameter (µm) | | # | ImageJ  diameter (µm) | | # | ImageJ  diameter (µm) | | | # | ImageJ diameter (µm) | | Mean |  | |  | |
| --- | --- | --- | --- | --- | --- | --- | --- | --- | --- | --- | --- | --- | --- | --- | --- | --- | --- |
| ***1*** | 29.3 |  | ***51*** | 17.1 |  | ***101*** | 18.2 |  | ***151*** | | 23.0 |  | **22.0** |  |  | |  |
| ***2*** | 18.4 |  | ***52*** | 15.6 |  | ***102*** | 15.7 |  | ***152*** | | 25.3 |  |  |  |  | |  |
| ***3*** | 30.9 |  | ***53*** | 18.4 |  | ***103*** | 19.5 |  | ***153*** | | 24.1 |  | St. Dev. |  |  | |  |
| ***4*** | 34.3 |  | ***54*** | 27.6 |  | ***104*** | 18.3 |  | ***154*** | | 20.9 |  | **6.0** |  |  | |  |
| ***5*** | 24.8 |  | ***55*** | 23.7 |  | ***105*** | 19.9 |  | ***155*** | | 18.6 |  |  |  |  | |  |
| ***6*** | 23.0 |  | ***56*** | 18.6 |  | ***106*** | 23.4 |  | ***156*** | | 20.5 |  |  |  |  | |  |
| ***7*** | 27.4 |  | ***57*** | 17.9 |  | ***107*** | 18.3 |  | ***157*** | | 24.2 |  | St Error |  |  | |  |
| ***8*** | 20.5 |  | ***58*** | 19.4 |  | ***108*** | 17.7 |  | ***158*** | | 25.3 |  | **0.4** |  |  | |  |
| ***9*** | 19.6 |  | ***59*** | 17.0 |  | ***109*** | 17.4 |  | ***159*** | | 27.4 |  |  |  |  | |  |
| ***10*** | 21.3 |  | ***60*** | 18.9 |  | ***110*** | 20.9 |  | ***160*** | | 23.2 |  |  |  |  | |  |
| ***11*** | 31.9 |  | ***61*** | 28.1 |  | ***111*** | 19.1 |  | ***161*** | | 22.7 |  |  |  |  | |  |
| ***12*** | 20.1 |  | ***62*** | 24.9 |  | ***112*** | 19.4 |  | ***162*** | | 30.3 |  |  |  |  | |  |
| ***13*** | 19.5 |  | ***63*** | 26.2 |  | ***113*** | 15.9 |  | ***163*** | | 29.2 |  |  |  |  | |  |
| ***14*** | 14.5 |  | ***64*** | 26.2 |  | ***114*** | 17.4 |  | ***164*** | | 19.7 |  |  |  |  | |  |
| ***15*** | 23.3 |  | ***65*** | 24.1 |  | ***115*** | 22.3 |  | ***165*** | | 21.7 |  |  |  |  | |  |
| ***16*** | 21.5 |  | ***66*** | 24.0 |  | ***116*** | 25.5 |  | ***166*** | | 20.1 |  |  |  |  | |  |
| ***17*** | 23.7 |  | ***67*** | 30.5 |  | ***117*** | 20.0 |  | ***167*** | | 23.0 |  |  |  |  | |  |
| ***18*** | 16.9 |  | ***68*** | 22.0 |  | ***118*** | 19.3 |  | ***168*** | | 23.5 |  |  |  |  | |  |
| ***19*** | 15.0 |  | ***69*** | 23.7 |  | ***119*** | 15.6 |  | ***169*** | | 20.7 |  |  |  |  | |  |
| ***20*** | 31.3 |  | ***70*** | 22.5 |  | ***120*** | 23.4 |  | ***170*** | | 25.8 |  |  |  |  | |  |
| ***21*** | 27.8 |  | ***71*** | 23.8 |  | ***121*** | 18.2 |  | ***171*** | | 16.8 |  |  |  |  | |  |
| ***22*** | 28.2 |  | ***72*** | 19.3 |  | ***122*** | 20.4 |  | ***172*** | | 23.7 |  |  |  |  | |  |
| ***23*** | 19.1 |  | ***73*** | 22.5 |  | ***123*** | 21.3 |  | ***173*** | | 22.6 |  |  |  |  | |  |
| ***24*** | 21.7 |  | ***74*** | 24.0 |  | ***124*** | 16.6 |  | ***174*** | | 23.0 |  |  |  |  | |  |
| ***25*** | 26.2 |  | ***75*** | 25.7 |  | ***125*** | 35.2 |  | ***175*** | | 17.3 |  |  |  |  | |  |
| ***26*** | 19.4 |  | ***76*** | 20.6 |  | ***126*** | 23.8 |  | ***176*** | | 19.5 |  |  |  |  | |  |
| ***27*** | 19.6 |  | ***77*** | 17.6 |  | ***127*** | 14.1 |  | ***177*** | | 19.0 |  |  |  |  | |  |
| ***28*** | 25.9 |  | ***78*** | 13.7 |  | ***128*** | 18.1 |  | ***178*** | | 27.4 |  |  |  |  | |  |
| ***29*** | 20.3 |  | ***79*** | 26.5 |  | ***129*** | 17.2 |  | ***179*** | | 19.5 |  |  |  |  | |  |
| ***30*** | 20.8 |  | ***80*** | 30.0 |  | ***130*** | 27.6 |  | ***180*** | | 21.4 |  |  |  |  | |  |
| ***31*** | 23.7 |  | ***81*** | 28.2 |  | ***131*** | 22.4 |  | ***181*** | | 18.7 |  |  |  |  | |  |
| ***32*** | 15.9 |  | ***82*** | 28.3 |  | ***132*** | 28.3 |  | ***182*** | | 25.0 |  |  |  |  | |  |
| ***33*** | 16.3 |  | ***83*** | 25.7 |  | ***133*** | 23.6 |  | ***183*** | | 21.9 |  |  |  |  | |  |
| ***34*** | 17.5 |  | ***84*** | 25.0 |  | ***134*** | 17.7 |  | ***184*** | | 17.7 |  |  |  |  | |  |
| ***35*** | 28.8 |  | ***85*** | 26.5 |  | ***135*** | 21.7 |  | ***185*** | | 29.9 |  |  |  |  | |  |
| ***36*** | 19.7 |  | ***86*** | 27.4 |  | ***136*** | 20.1 |  | ***186*** | | 22.1 |  |  |  |  | |  |
| ***37*** | 16.0 |  | ***87*** | 31.6 |  | ***137*** | 22.3 |  | ***187*** | | 17.8 |  |  |  |  | |  |
| ***38*** | 16.3 |  | ***88*** | 28.6 |  | ***138*** | 21.0 |  | ***188*** | | 18.0 |  |  |  |  | |  |
| ***39*** | 27.4 |  | ***89*** | 29.1 |  | ***139*** | 23.4 |  | ***189*** | | 21.8 |  |  |  |  | |  |
| ***40*** | 22.1 |  | ***90*** | 27.2 |  | ***140*** | 20.2 |  | ***190*** | | 19.8 |  |  |  |  | |  |
| ***41*** | 36.3 |  | ***91*** | 40.7 |  | ***141*** | 23.1 |  | ***191*** | | 26.1 |  |  |  |  | |  |
| ***42*** | 24.2 |  | ***92*** | 24.9 |  | ***142*** | 23.3 |  | ***192*** | | 23.9 |  |  |  |  | |  |
| ***43*** | 33.1 |  | ***93*** | 31.2 |  | ***143*** | 26.5 |  | ***193*** | | 20.5 |  |  |  |  | |  |
| ***44*** | 15.6 |  | ***94*** | 23.0 |  | ***144*** | 27.4 |  | ***194*** | | 25.1 |  |  |  |  | |  |
| ***45*** | 14.2 |  | ***95*** | 29.6 |  | ***145*** | 17.7 |  | ***195*** | | 19.2 |  |  |  |  | |  |
| ***46*** | 9.9 |  | ***96*** | 30.6 |  | ***146*** | 24.5 |  | ***196*** | | 17.3 |  |  |  |  | |  |
| ***47*** | 13.4 |  | ***97*** | 28.5 |  | ***147*** | 19.4 |  | ***197*** | | 9.7 |  |  |  |  | |  |
| ***48*** | 15.2 |  | ***98*** | 15.9 |  | ***148*** | 23.4 |  | ***198*** | | 14.3 |  |  |  |  | |  |
| ***49*** | 21.6 |  | ***99*** | 19.0 |  | ***149*** | 32.3 |  | ***199*** | | 27.0 |  |  |  |  | |  |
| ***50*** | 14.6 |  | ***100*** | 27.4 |  | ***150*** | 26.3 |  | ***200*** | | 21.6 |  |  |  |  | |  |

Individual diameter measurements for particle size evaluation (n = 200) performed by ImageJ.

**Figure S4.**

 Energy Dispersive X-Ray (EDX) analysis of an individual e-ink microcapsule (EIMC): (A) relatively coarse surface area [Z] and inner side of the cracked surface [W]; (B) typical corresponding EDX spectrum associated with [W]. Observation conditions: Accelerating Voltage = 15 kV, Magnification = x1500, Working Distance = 8.8 mm, Emission Current = 59.1 mA, Filament Current=1850 mA.

**Table S2.**

**Summary results**

| Element | Atomic %  [Z] | Atomic % [W] |
| --- | --- | --- |
| Carbon | 53.5 | 29.9 |
| Oxygen | 28.5 | 16.9 |
| **Titanium** | 16.9 | 52.3 |
| Gold | 1.1 | 0.9 |

Summary results of EDX analysis associated with the selected areas of the EIMC in Figure S2 (Figure 2C in the manuscript). A quantitative range of the elemental atomic percentage is provided.

**Figure S5.**

Surface electrokinetic potential (SEKP) of unmodified (blue) and surface-modified TiO_2_ NPs (red) following deposition of MMA-EGDMA copolymer. The average SEKP becomes more negative (-49.3 ± 0.6 mV) after modification from -23.6 ± 0.9 mV, indicating enhanced surface charge and colloidal stability by increasing electrostatic repulsion, reducing NP agglomeration, and optimising dispersion in the electrophoretic medium.
